# Reinforcement of Novel PLA/17-4 PH Stainless Steel Hybrid Structures Fabricated by FDM: The Effects of Layer Configuration, Infill Density and Pattern

**DOI:** 10.3390/polym18060672

**Published:** 2026-03-10

**Authors:** Ramazan Ötüken, Cem Alparslan, Muhammed Furkan Erhan, Şenol Bayraktar

**Affiliations:** 1 Department of Electronic Automation, Vocational School of Technical Sciences, Recep Tayyip Erdoğan University, Rize 53100, Türkiye; ramazan.otu@erdogan.edu.tr; 2 Department of Mechanical Engineering, Faculty of Engineering and Architecture, Recep Tayyip Erdogan University, Rize 53100, Türkiye; cem.alparslan@erdogan.edu.tr; 3 FDM and Metallography Laboratory, Department of Mechanical Engineering, Recep Tayyip Erdogan University, Rize 53100, Türkiye; 4 Department of Manufacturing Engineering, Faculty of Technology, Gazi University, Ankara 06560, Türkiye; furkanerhan@gazi.edu.tr

**Keywords:** FDM, hybrid additive manufacturing, layer configuration, infill density, infill pattern, mechanical properties, RSM

## Abstract

Fused deposition modeling/fused filament fabrication (FDM/FFF) enables architectural tailoring of mechanical response through layer configuration and multi-material manufacturing strategies. However, the combined effects of layer arrangement, infill ratio, and packing geometry in polymer–metal hybrid structures and interfacial load transfer mechanisms are still not sufficiently elucidated. In this study, the tensile behavior of single- and multi-material structures produced using PLA and 17-4 PH stainless steel filaments was systematically investigated. A total of 24 experimental parameter sets were created with four-layer configurations (PLA, 17-4 PH, PLA/17-4 PH/PLA, and 17-4 PH/PLA/17-4 PH), three infill ratios (20%, 60%, and 100%), and two packing patterns (linear and hexagonal); the samples were tested according to the ASTM D638 standard. Mechanical data were modeled using Response Surface Methodology (RSM) and ANOVA, and the developed regression models showed high accuracy (R^2^ > 0.95). The findings showed that tensile and yield strength are primarily controlled by the layer arrangement, while infill ratio and infill pattern have a secondary effect. The highest strength was measured in 100% infill linear PLA samples (≈10.35 MPa), and the lowest value was measured in 17-4 PH “green part” samples without sintering (≈0.92 MPa). Hybrid structures exhibited intermediate performance in the range of 2.9–4.9 MPa. ANOVA results showed that the majority of the mechanical variance was explained by the layer arrangement (70–85% contribution), while infill ratio and infill pattern had a secondary effect. Fracture surface analyses showed that high performance was associated with homogeneous filament fusion and low porosity; Studies have confirmed that poor performance is associated with delamination and interfacial separation.

## 1. Introduction

Additive manufacturing (AM) technologies have fundamentally changed modern production paradigms thanks to their ability to create three-dimensional components directly from digital designs, and have attracted significant attention in both academic and industrial fields in recent years. These technologies stand out as a strategic alternative to traditional methods such as CNC machining, injection molding, forging, and casting due to their advantages such as the production of complex geometries, shorter lead times, lower initial investment costs, and minimal material waste [1,2]. Initially limited to prototyping, AM applications have evolved to enable the production of end-use components in sectors such as aerospace, healthcare, automotive, and defense [3]. AM technologies are divided into subclasses based on polymer, metal, ceramic, and composite, depending on the type of material used and the bonding mechanism [4,5,6]. Within these subcategories, AM with metals stands out due to its superiority, particularly in applications requiring high mechanical strength, thermal stability, and resistance to harsh service conditions. Increased performance expectations in high-value-added sectors such as aerospace, defense, energy, and medical have made the integration of AM with metal materials a strategic research area.

Although techniques such as Selective Laser Melting (SLM), Electron Beam Melting (EBM), and Powder Bed Fusion (PBF) dominate the market in the Metal AM field, the high operating costs and safety risks associated with powder management of these systems have led researchers to seek more accessible methods [7]. In this context, Material Extrusion Additive Manufacturing (MEAM), or Fused Filament Manufacturing (FFF) as it is commonly known, initially developed for thermoplastic processing, stands out as a low-cost and office-friendly solution for the production of metal components [8,9]. The Metal FFF process is based on the extrusion of a composite filament consisting of metal powder and a polymer binder, layer by layer, followed by binder removal and sintering steps [10]. The type of polymeric binder systems and filament architecture used in this process indirectly affect not only the forming capability but also the density and mechanical integrity of the final part. Therefore, filament design approaches, starting with materials such as ABS (Arylonitrile butadiene styrene), ASA (Acrylonitrile styrene acrylate), PC (Polycarbonates), PETG (Polyethylene Terephthalate Glycol), PA (Polyamide), and PLA (Polylactic acid), commonly used in metal-based additive manufacturing (MFF), have evolved over time into reinforced and functional composite structures to meet higher performance requirements. The performance improvements provided by reinforcements such as carbon fiber and graphene in polymer-based FFF processes, and by 17-4 PH stainless steel in metal-based FFF applications, have similarly paved the way for material development studies aimed at improving the engineering properties of metal FFF filaments [11].

Among the alloys commonly preferred in metal FFF-based heat treatment applications within composite materials, 17-4 PH stainless steel is one of the most widely used martensitic stainless steels in the aerospace, nuclear, petrochemical, automotive, turbine shaft, and marine industries due to its high strength, toughness, wear and corrosion resistance, and suitability for heat treatment through precipitation hardening [3,12,13]. However, the high hardness and strength of 17-4 PH stainless steel make it difficult to machine using conventional machining and forming methods. This situation both increases production time and significantly increases costs. At this point, the metal FFF method offers a significant advantage by providing the potential to produce complex and functional geometries of difficult-to-machine alloys like 17-4 PH in a near-net-shape form [14].

Studies in the literature on the production of 17-4 PH stainless steel using the metal FFF method clearly demonstrate the decisive role of production parameters on microstructure, mechanical performance, and environmental resistance. In particular, the effects of pressing direction and layer placement on anisotropic behavior have been emphasized in many studies. Alkindi et al. recommended pressing angles between 0° and 10° to ensure optimum tensile performance and structural integrity [15]. Tasci and Yılmaz determined that among different infill patterns (Solid, Gyroid, and Triangular), the plain infill pattern provided the highest hardness, strength, transverse fracture strength (TRS), and relative density (96.4%), as well as a low wear rate [16]. However, the corrosion behavior of 17-4 PH components produced with metal FFF is significantly affected by the manufacturing conditions, not only in terms of their mechanical properties. Mwema et al. observed that temperature fluctuations in the operating environment affect the austenite phase in the microstructure, leading to deterioration in corrosion resistance [17]. Liew et al. investigated the microstructural and mechanical performance of samples after printing, sintering, and post-processing [7]. In the study, which demonstrated the effects of anisotropic behavior of sintered samples depending on the printing direction, samples produced perpendicular to the printing direction (90°) showed superior mechanical properties, exhibiting a 28% increase in tensile strength and approximately an eightfold improvement in elongation to fracture (a ductility indicator) compared to those produced parallel to the printing direction (0°). The effects of heat treatment and sintering conditions have also been discussed in detail in the literature. Romero et al. observed that after H900 heat treatment, the samples reached a relative density of 96.20% and the pore structure consisted of isolated, rounded, and minimal voids generally located at the layer interfaces. Furthermore, it was reported that heat treatment improved the material properties in terms of hardness, Young’s modulus, ultimate tensile strength, yield strength, and plastic strain [3]. The research results in the study conducted by Ahmad revealed that atmosphere selection is decisive in densification and mechanical behavior. It was determined that sintering processes carried out in a vacuum environment significantly increased tensile strength and hardness, while argon atmospheres improved the impact resistance and energy absorption capacity of the material [12]. Porosity, one of the most important factors limiting structural reliability in metal FFF parts, has been addressed through both experimental and numerical studies. Porrang et al. studied the simulation of porosity within the material, which negatively affects material strength, using the Gurson-Tvergaard-Needleman (GTN) damage model [18]. They demonstrated that the numerical calculations of the finite element model were in good agreement with experimental data. The presence of porosity in metal FFF parts leads to inconsistencies in mechanical properties and potentially affects component performance by causing significant complications in terms of structural reliability. Therefore, focusing on how porosity affects material behavior, Porrang et al. conducted experimental analyses to measure the porosity of FFF. These analyses provided reasonable accuracy in predicting stress–strain behavior in both elastic and plastic regions using a modeling approach that directly incorporates porosity through element extraction in a finite element model [19]. When these studies are considered together, it is clearly seen that the mechanical and environmental performance of 17-4 PH stainless steel components produced by the metal FFF method is determined by the interaction of numerous process parameters such as printing direction, layer arrangement, infill pattern, sintering atmosphere, and microstructural porosity. However, it is noteworthy that in the current literature, these parameters are mostly considered individually or in limited combinations; and that studies systematically evaluating the combined effects of different production parameters in the context of microstructure-mechanical property relationships are limited. Furthermore, the role of layer architecture and filling strategies on porosity formation and the resulting mechanical consistency remains an important research area that needs to be clarified in terms of metal FFF processes.

While pure polymeric materials such as PLA can exhibit relatively high tensile strength under optimized conditions, their structural behavior remains limited in terms of multi-material integration, stiffness modulation, and architectural load transfer when combined with dissimilar material systems. Hybrid polymer–metal architectures offer an alternative strategy to investigate how dissimilar material layers interact mechanically within a single FDM process. In the present study, the PLA/17-4 PH combination was employed not to immediately surpass the tensile strength of pure PLA, but to examine how green-state metal–polymer layering influences structural response, stress distribution, and interfacial behavior. Therefore, the contribution of this work lies in evaluating architectural feasibility and interlayer mechanics in unsintered hybrid FDM systems rather than direct strength enhancement.

This study aims to systematically investigate the mechanical behavior of multi-material specimens with different infill ratios, infill patterns, and layer arrangements using the FDM-based AM method. In this context, four different layer configurations were designed using 20%, 60%, and 100% infill ratios and two different infill patterns (linear and hexagonal). The first specimen was made entirely of PLA material, the second entirely of 17-4 PH stainless steel filament; the third specimen was a PLA–17-4 PH–PLA configuration, and the fourth specimen was a 17-4 PH–PLA–17-4 PH multilayer composite structure. Tensile tests were performed to determine elastic modulus, yield strength, ultimate tensile strength, and elongation at fracture. Response Surface Methodology (RSM) was used to statistically evaluate individual and interactive effects of infill ratio, infill pattern, and layer arrangement.

In addition to mechanical testing, fracture surfaces were examined using optical microscopy to evaluate interlayer bonding quality and damage mechanisms. This integrated experimental–statistical–microstructural framework enables systematic evaluation of architectural load transfer and interfacial behavior in unsintered metal–polymer FDM systems. From a scientific perspective, combining green-state 17-4 PH stainless steel with PLA allows isolation of layer arrangement effects from post-sintering densification phenomena, thereby providing insight into stiffness mismatch, stress redistribution, and failure evolution in dissimilar material stacking. The significance of this work lies in advancing understanding of interfacial mechanics and structural feasibility within hybrid FDM architectures and contributing to the design-oriented development of multi-material FDM systems.

## 2. Material and Method

**Figure 1:**
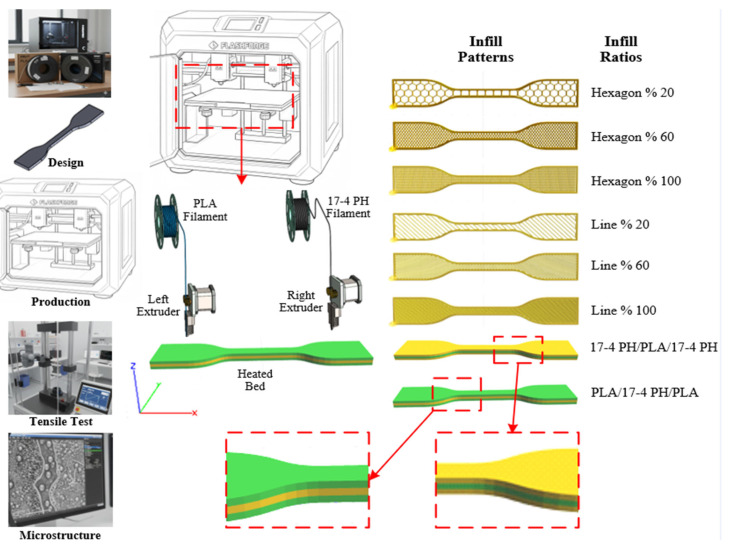
Workflow schema.

In this experimental study, commercially available PLA and 17-4 PH stainless steel-based filaments were used. Standard test specimens were produced to investigate the mechanical effects of the filaments on composite layered structures. The experimental process applied to the test specimens consisted of five main stages: material procurement, specimen design, FDM-based production, mechanical testing, and microstructural characterization. The general workflow diagram of the study is presented in Figure 1.

### 2.1. Material and Filament Characteristics

In the studies, commercial filaments of two different classes, PLA and 17-4 PH, with a diameter of 1.75 mm were used. PLA filament was chosen as the reference material in FDM processes due to its low thermal expansion coefficient and high dimensional stability. Although PLA exhibits brittle fracture behavior, it shows high tensile strength and stiffness characteristics among polymers. The PLA-based filament used in the study was obtained from the ESUN brand [20]. Although PLA offers high rigidity in room temperature applications, it rapidly loses its strength at temperatures above 60 °C. In contrast, 17-4 PH exhibits high corrosion resistance and high toughness under extreme loads. In the composite configurations used in this study, the combined effects (synergistic effects) of PLA’s lightness and ease of processing with 17-4 PH’s structural integrity and stiffness were investigated.

17-4 PH Stainless Steel (Ultrafuse^®^, Ludwigshafen, Germany) used for composite production: Developed by BASF Forward AM to optimize the metal FFF process. The filament architecture features a catalytic binder system designed to maintain dimensional stability and ensure fluidity during extrusion. The catalytic binder system is a proprietary thermoplastic binder system that governs the mechanical behavior of the filament prior to sintering. The interfacial adhesion between this binder system and PLA during multi-material deposition is mainly achieved through thermal interlayer fusion and mechanical interlocking rather than chemical bonding, due to differences in polymer chemistry. The 17-4 PH stainless steel filament contains 80% metal powder, increasing the thermal conductivity of the material and providing high density to the manufactured parts, even in its raw (green) state. The basic mechanical properties of the filaments declared by the manufacturers are presented in Table 1. The 17-4 PH metal filament was handled with special care due to its inherent brittleness in the green state. The filament spool was stored in a sealed container with desiccant at room temperature prior to printing. No additional thermal drying procedure was applied before use, as the filament was supplied in vacuum-sealed packaging and used shortly after opening. During printing, careful feeding and minimal bending were ensured to prevent filament breakage and maintain consistent extrusion.

### 2.2. Experimental Design and FDM Printing Parameters

The samples were produced using a Flashforge Creator 3 desktop FDM printer (Zhejiang, China) with industrial-grade precision. The main reason for choosing this system is its Independent Dual Extruder (IDEX) technology. IDEX technology allows two materials with completely different thermal and mechanical properties, such as PLA and 17-4 PH, to be printed on the same build plate to form a composite structure without cross-contamination [22].

The printer features high-temperature extruders capable of reaching temperatures up to 300 °C. Considering the high abrasiveness of 17-4 PH metal filament, 0.4 mm diameter hardened steel nozzles were used instead of standard brass nozzles. Furthermore, to prevent thermal stresses and delamination during printing, the printer’s enclosed cabinet structure and 120 °C heated bed was actively utilized. The cabinet temperature was kept constant, especially since the cooling rate of metal-containing layers is critical for structural integrity. The device also enabled the precise creation of complex fill patterns (hexagonal and linear). The optimized process parameters used in the printing process are presented in Table 2.

Multi-material structures, which form the focus of this study, were produced in four different configurations based on the parameters in Table 2. In the hybrid configurations, the notations PLA–17-4 PH–PLA and 17-4 PH–PLA–17-4 PH indicate a three-layer stacking sequence along the build (thickness) direction. The exact layer thicknesses are defined in Table 2 as 1.3 mm for the initial (bottom) layer and 1.35 mm for both the middle and top layers (total thickness ≈ 4.0 mm). In the composite samples (PLA, 17-4 PH, PLA-17-4 PH-PLA, and 17-4 PH-PLA-17-4 PH), the inter-extruder calibration (Z-offset) was precisely adjusted to improve adhesion quality at the transitions between layers. The samples were sliced using Flashprint 5.6.0 slicing software, with the first layers at 0.25 mm and the top layers at 0.15 mm. For each configuration, a total of 24 different experimental parameters were determined using 20, 60, and 100% infill ratios and linear and hexagonal infill patterns. Three test samples were produced for each experimental parameter. The arithmetic mean of the obtained results was calculated, and the final mechanical properties were determined.

### 2.3. Mechanical Testing

**Figure 2:**
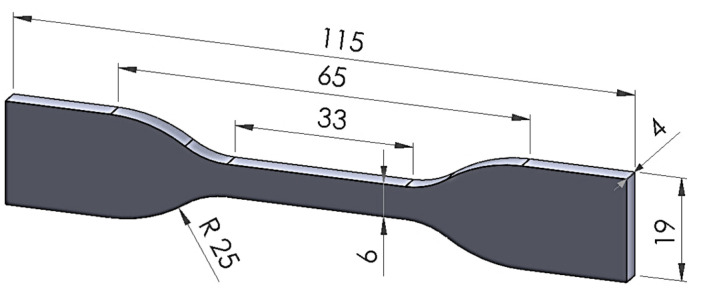
Shape and dimension of specimens for tensile test (Dimensions are in mm).

The tensile specimen for tensile testing was prepared according to ASTM D638-14 standard Type IV (Figure 2) [23,24].

**Figure 3:**
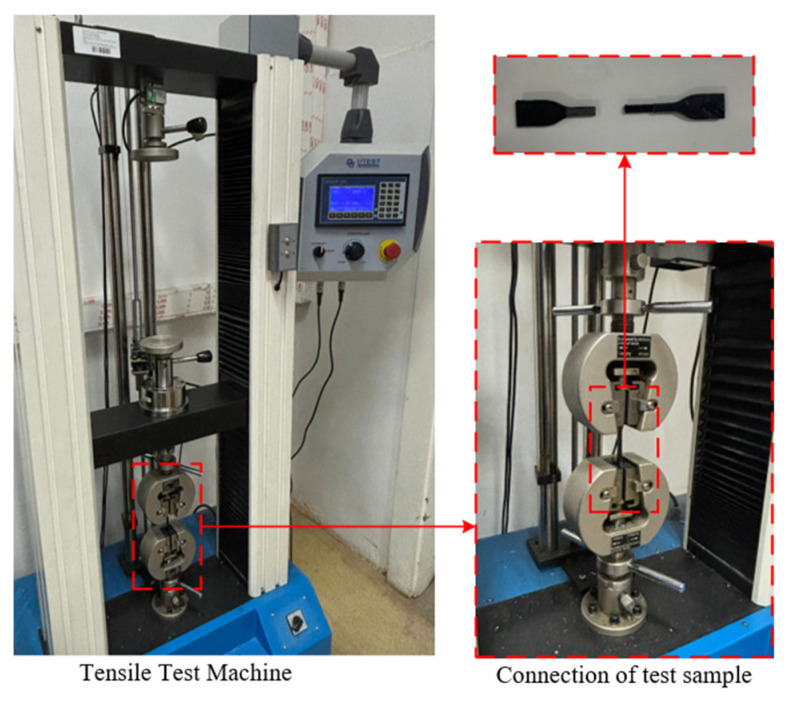
UTEST PROFI X6 tensile test machine.

Tensile tests of PLA, 17-4 PH, PLA-17-4 PH-PLA and 17-4 PH-PLA-17-4 PH samples were performed on a Utest-Profi X6 tensile testing machine at a constant loading speed of 5 mm/min (Figure 3).

Tensile tests were performed on each sample according to four different configurations, infill patterns, and layer thicknesses. Yield strength (N/mm^2^), tensile strength (N/mm^2^), and elongation to fracture (%) values were obtained from the tensile tests. To minimize the margin of error, at least three samples were tested for each parameter set, and the average values were recorded.

### 2.4. Fracture Surface Characterization

To evaluate fracture mechanisms and interlayer adhesion quality, the fracture surfaces of the samples were directly subjected to macro-level analyses. Following mechanical tests, no sectioning, polishing, or additional surface preparation was applied to the samples; characterization was performed while preserving the natural surfaces formed during fracture. This approach allowed for the observation of the damage morphology developed during the fracture process in its original form and eliminated artifacts that might arise from sample preparation.

Fracture surfaces were examined at different magnification ratios using a Dino-Lite premier digital microscope (AnMo Electronics Corporation, Taiwan) and high-resolution images were recorded. The examinations included a detailed analysis of surface topography, interlayer delamination, interface ruptures, void and pore formation, filament traces, crack initiation zones, and crack propagation paths. These findings formed the basis for a qualitative comparison of fracture behavior depending on the manufacturing direction and for interpreting the relationship between mechanical performance and interlayer structural integrity. The microstructural observations provided complementary evidence for understanding the effects of manufacturing parameters and printing direction on damage mechanisms and supported the interpretation of mechanical test results.

### 2.5. Response Surface Methodology

The RSM method was used to quantitatively model the effects of production parameters on mechanical properties, determine the interactions between factors, and predict optimum production conditions [25,26]. All statistical analyses, modeling, and optimization processes were performed using Minitab 19 statistical software.

Because the study includes both continuous and categorical variables, a quadratic mixed-factor response surface approach based on the General Linear Model (GLM) was preferred over classical central composite (CCD) or Box–Behnken designs. This method allows for the evaluation of linear and quadratic effects of continuous variables and their interactions with categorical variables within the same model.

Independent variables are defined as follows:

- Infill percentage: 20, 60, and 100% (continuous variable);
- Infill pattern: Linear and Hexagonal (categorical variable);
- Material configuration: PLA, 17-4 PH, PLA-17-4 PH-PLA, and 17-4 PH-PLA-17-4 PH (categorical variable).

The response variables were defined as tensile strength (TS), yield strength (YS), and elongation to fracture.

A second-order regression model was constructed for each response in the following general form:

(1) $$y=\beta_{0}+\beta_{1}+\beta_{2}x^{2}+\beta_{3}\left(Material\right)+\;\beta_{4}\left(Pattern\right)+\;\beta_{5}\left(x\times Material\right)+\;\beta_{6}\left(x\times Pattern\right)+\;\beta_{7}\left(Materail\times Pattern\right)+\;\varepsilon \;$$

Here;

y: Response (UTS, YS or EL).
x: Infill (%).
Material: Layer arrangement (Categorical factor).
Pattern: Infill pattern (Categorical factor).
x^2^: Quadratic (Curvature) term.
ε: Error term.

The following analyses were performed during the statistical evaluation process:

- Model significance: The statistical significance of regression and interaction terms was evaluated using analysis of variance (ANOVA), and a significance level of *p* < 0.05 was used.
- Model fit: The explanatory power of the models on experimental data was examined using the coefficient of determination (R^2^) and the adjusted coefficient of determination (Adj-R^2^).
- Residual analyses: Residual plots were evaluated for normality, constant variance, and independence checks to verify the model assumptions.
- Response surface visualization: Contour and three-dimensional surface plots were created to show the combined effects of the factors.
- Optimization: Optimal parameter combinations that maximize mechanical performance were determined using Minitab’s “Response Optimizer” tool.

Thanks to this holistic approach, the mechanical behavior of composite structures has been mathematically modeled, the interactions between factors have been quantitatively and graphically revealed, and the most suitable production parameters have been reliably predicted.

## 3. Results and Discussion

### 3.1. Mechanical Properties of Single- and Multi-Material FDM Structures

The tensile behavior of specimens produced with single and multi-material FDM was systematically evaluated under the parameters of infill ratio (20, 60 and 100%) and infill pattern (linear and hexagonal). The literature reports that the mechanical performance of FDM parts is strongly controlled by infill density and internal geometry; as density increases, tensile strength increases due to the decrease in void ratio and the increase in load transfer continuity [27,28,29].

In hybrid FDM structures, infill rate is a critical design parameter because it directly controls the effective load-bearing area, internal stress distribution, and stiffness compatibility between dissimilar materials. Since the present study focuses on mechanically integrated green parts, the mechanical response is governed not only by interfacial bonding but also by the macro-scale structural continuity provided by the infill architecture. Adjusting the infill ratio allows systematic evaluation of load transfer efficiency between polymer and metal-containing regions by modifying internal support density and deformation pathways. Therefore, infill rate was selected as the primary variable to isolate the structural contribution of hybrid architecture under identical processing conditions.

**Figure 4:**
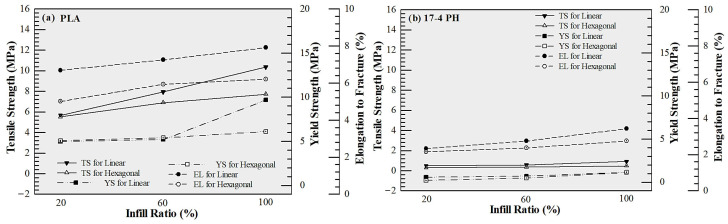
Comparison of tensile strength, yield strength, and elongation to fracture of test specimens, (**a**) PLA and (**b**) 17-4 PH materials.

The results obtained showed that PLA samples exhibited the highest mechanical performance. Under linear infill, tensile strength was measured as 5.667, 7.933, and 10.347 N/mm^2^ at 20%, 60%, and 100% infill, respectively; yield strength increased from 3.792 to 6.958 N/mm^2^ (Figure 4a). Although the tensile strength increased with infill ratio, the measured value for the 100% infill PLA specimen (~10 MPa) is lower than the typical range reported in the literature for FDM-printed PLA. This discrepancy can be attributed to the process-sensitive nature of FDM fabrication. It should be noted that selecting 100% infill does not necessarily imply a fully dense structure; variations in extrusion flow, raster bonding, and cooling conditions may lead to micro-voids and incomplete interlayer fusion, which can significantly reduce load-bearing capacity. Furthermore, the anisotropic structure inherent to layer-by-layer fabrication may promote interlayer fracture under tensile loading, particularly when interfacial bonding is not fully optimized. In addition, specimen geometry and testing conditions may also contribute to early failure behavior. Therefore, the relatively low tensile strength observed in this study is likely the result of combined process-induced porosity, interlayer bonding limitations, and testing-related factors rather than a single dominant cause. Despite the relatively low absolute strength level, the increasing trend with infill ratio can be attributed to improved material continuity and reduced internal void content, consistent with the density-strength correlation reported in the literature [30]. In hexagonal infill, the maximum strength remained at 7.722 N/mm^2^, yielding lower results compared to linear geometry (Figure 4a). This can be explained by the advantage of linear raster, which creates a more direct load path in the direction of the tensile load [31]. In contrast, samples produced from 17-4 PH filaments showed the lowest strength values (Figure 4b) (Maximum 0.917 N/mm^2^). In this study, no debonding or sintering process was applied to the metal filaments, and the samples were tested as “green parts”. It is clearly stated in the literature that in metal powder-polymer binder-based FFF/MEX filaments, the mechanical behavior before sintering is largely controlled by the polymer binder, and the strength is limited due to the weak interlayer bonding [32,33,34]. Therefore, the observed low tensile values reflect the behavior of the binder-dominant composite structure, not the true metallic properties [35,36].

**Figure 5:**
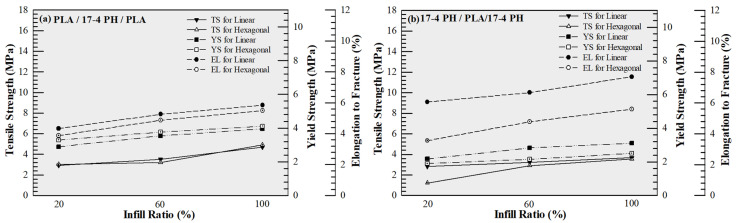
Comparison of tensile strength, yield strength, and elongation to fracture of test specimens, (**a**) PLA/17-4 PH/PLA and (**b**) 17-4 PH/PLA/17-4 PH materials.

Multi-material hybrid structures exhibited intermediate values among single materials in terms of mechanical performance. The highest tensile strength was determined as 4.917 N/mm^2^ in the PLA–17-4 PH–PLA configuration (Figure 5a), and 2.905 N/mm^2^ in the 17-4 PH–PLA–17-4 PH structure (Figure 5b). The decrease in strength in hybrid structures compared to pure PLA can be attributed to the incompatibility of stiffness/strain capacity of different materials, leading to stress discontinuities and stress concentrations at the interfaces, making these regions critical for crack initiation. It has been reported that in multi-material FFF/FDM systems, interfacial bonding directly controls the overall mechanical performance; when the interface is weak, damage mostly starts at the interface and progresses as delamination/separation, thus significantly limiting tensile strength and elongation to fracture [37,38,39,40]. In addition to strength considerations, the hybrid configurations enable local stiffness tailoring while reducing the overall volumetric contribution of high-density metal regions. Since the density of 17-4 PH (7.6 g/cm^3^) is significantly higher than that of PLA (1.2 g/cm^3^), strategic placement of metal layers allows structural reinforcement in selected regions without producing a fully metal-dense component. Therefore, the hybrid approach should be interpreted in the context of stiffness-to-weight balance and architectural design flexibility rather than absolute tensile strength maximization.

The infill density generally increased the strength in all configurations; however, the increase was not linear in hybrid structures. This indicates that not only the cross-sectional area but also the load transfer capacity at the interface is a limiting factor. The fracture elongation results also support this interpretation. PLA specimens exhibited more ductile behavior, while earlier fracture occurred in metal filament and multi-material structures due to interfacial separation. Overall, the findings reveal that the mechanical behavior in multi-material structures produced with FDM is controlled not only by infill and pattern parameters but also by interfacial bonding quality and stress transfer mechanisms. These mechanical trends are morphologically confirmed by fracture surface analyses presented in the next section. These observations are consistent with previously reported studies on polymer FDM and multi-material additive manufacturing systems, confirming that interfacial bonding and load transfer continuity are key factors governing mechanical performance and validating the present findings.

### 3.2. Fracture Surface Analysis and Failure Mechanisms

**Figure 6:**
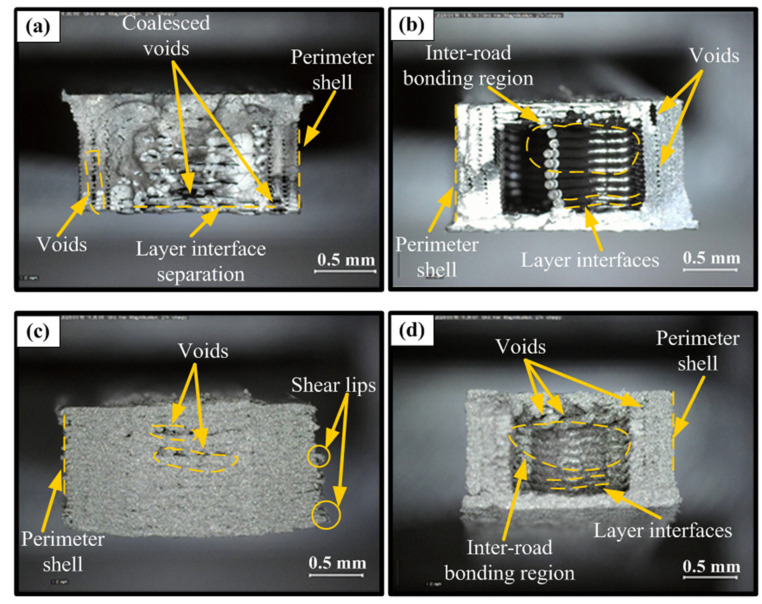
Fractured surfaces of FDM tensile test specimens, (**a**) PLA (Best mechanical properties), (**b**) PLA (Worst mechanical properties), (**c**) 17-4 PH (Green/best mechanical properties), and (**d**) 17-4 PH (Green/Worst mechanical properties).

Fracture surface analyses revealed that the differences in mechanical performance of single- and multi-material FDM structures have direct microstructural origins. PLA specimens exhibiting the highest tensile strength (Figure 6a) showed a more compact and continuous morphology, strong raster fusion, and effective load-carrying of the perimeter shell, while only a limited number of coalesced voids and local layer interface separations were detected. This is consistent with increased inter-road diffusion bonding and more homogeneous stress distribution at high infill ratios, confirming the density-strength correlation reported in the literature [29,30]. In contrast, the lowest-performing PLA samples showed distinct layer boundaries, poor filament fusion, and macroscopic void clusters, indicating delamination-controlled crack propagation initiated at the layer interfaces, resulting in early and brittle fracture behavior due to reduced effective cross-sectional area and stress concentrations (Figure 6b) [28].

In the unsintered “green part” samples produced from metal-based 17-4 PH filaments, fracture surfaces were characterized by a binder-dominant granular structure, particle pull-out, and cohesion loss rather than metallic ductile fracture; the load being carried primarily by the polymer binder limited mechanical strength. The best 17-4 PH configuration, with relatively low porosity, exhibited more integral fracture (Figure 6c), while in the weakest case, pronounced clusters of porosity, poor inter-road bonding, and layer discontinuities accelerated crack propagation, leading to premature failure (Figure 6d). These findings are consistent with the reported behavior of porosity and interfacial defects as sources of stress concentration in the metal FFF literature [32,33,36]. Overall, fracture surface findings indicate that mechanical performance in FDM structures is primarily determined by interlayer adhesion and porosity control. Furthermore, the combination of low porosity and high fusion provides high strength, while interfacial discontinuities lead to premature delamination and brittle fracture.

**Figure 7:**
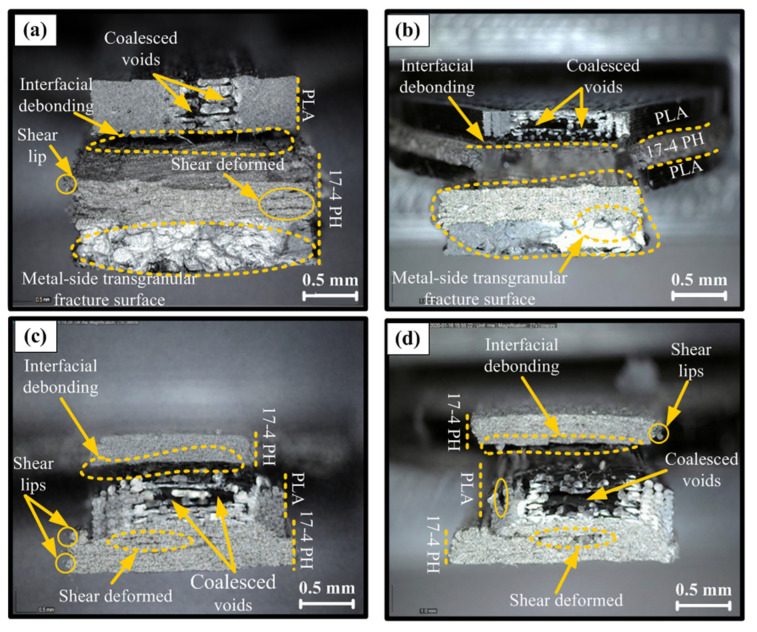
Fractured surfaces of multi-material FDM tensile test specimens: (**a**) PLA/17-4 PH/PLA (Best mechanical properties); (**b**) PLA/17-4 PH/PLA (Worst mechanical properties); (**c**) 17-4 PH/PLA/17-4 PH (Best mechanical properties); and (**d**) 17-4 PH/PLA/17-4 PH (Worst mechanical properties).

The fracture surfaces of multi-material sandwich configurations clearly demonstrate that mechanical performance is primarily controlled by interfacial adhesion and porosity. In the highest-strength PLA/17-4 PH/PLA structure (Figure 7a), interlayer continuity was largely preserved, a more compact morphology developed except for limited interfacial debonding and localized coalescing voids, and a more integral (transgranular-like) fracture was observed in the metal core. This indicates that the outer PLA layers effectively carry the load, homogenizing the stress distribution and reducing stress concentrations at the interface. In contrast, the lowest-performing PLA/17-4 PH/PLA sample (Figure 7b) showed significant interfacial separation, void clustering, and delamination propagating along the layer, indicating that cracks originated and propagated directly from the PLA/metal interface. This type of adhesion loss is frequently reported in the literature as a primary cause of early failure in multi-material FDM structures [37,39]. A similar trend was observed in the 17-4 PH/PLA/17-4 PH configuration; while the relatively better performing sample (Figure 7c) showed limited debonding and local shear lips with partial plastic deformation, in the weakest case (Figure 7d), high porosity, weak inter-road bonding, and large-scale interface separation accelerated crack propagation, leading to brittleness and premature fracture. It is known that in metal FFF “green part” structures, binder-dominant behavior and high porosity limit load transfer, and interface discontinuities act as sources of stress concentration [32,41]. Overall, the fracture surface findings confirm that in hybrid structures, mechanical strength is determined not only by infill ratio or layer arrangement, but critically by the quality of PLA/metal interface bonding and porosity control.

**Figure 8:**
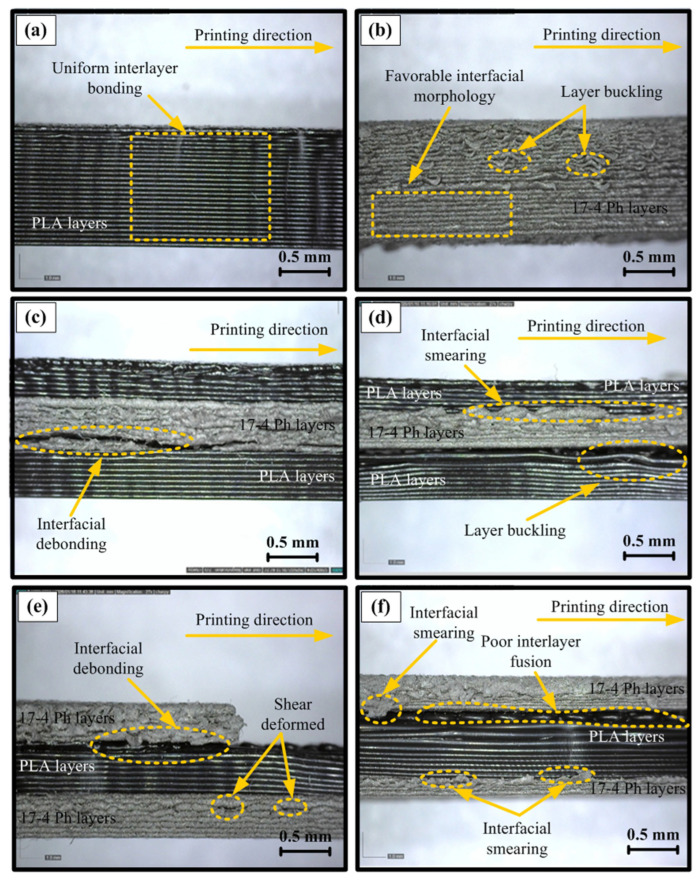
Cross-sectional micrographs showing interlayer morphology of FDM specimens, (**a**) PLA with uniform interlayer bonding, (**b**) 17-4 PH with local layer buckling, (**c**) PLA/17-4 PH/PLA with interfacial debonding, (**d**) PLA/17-4 PH/PLA with interfacial smearing and buckling, (**e**) 17-4 PH/PLA/17-4 PH with interfacial debonding, and (**f**) 17-4 PH/PLA/17-4 PH with interfacial smearing and buckling.

Cross-sectional (side profile) images clearly revealed that the macroscopic mechanical performance of the samples is directly determined by the layer architecture, interfacial fusion quality, and porosity distribution. In the pure PLA sample exhibiting the highest strength (Figure 8a), it was observed that the filaments were stacked regularly and parallel, a continuous and void-free morphology was formed between the layers, and the inter-raster boundaries were largely fused, exhibiting a singular mass behavior. This structure indicates that strong interlayer adhesion is achieved through sufficient thermal diffusion and mutual penetration of polymer chains, and that the load can be transferred seamlessly between the layers. In contrast, in the lowest-performing PLA sample (Figure 8b), layer buckling, irregular surface topography, and local fusion deficiencies were evident. It is shown that these geometric discontinuities facilitate crack initiation by creating stress concentration zones and increasing the tendency for delamination. Similar morphologies have been reported in the literature to be associated with early failure under conditions of low infill and insufficient bonding [42,43]. The observed interfacial debonding is consistent with the expected behavior of multi-material green parts and reflects limited interfacial compatibility rather than experimental deficiency. The comparison of different layer sequences provides insight into stress redistribution mechanisms in hybrid architectures despite this behavior.

In the best-case scenario (Figure 8c) of the hybrid PLA/17-4 PH/PLA configuration, relative contact continuity was maintained in the polymer–metal transition zone and limited debonding was observed, while in the weakest sample (Figure 8d), significant interfacial separation and loss of layer stability disrupted load transfer, causing cracks to propagate directly along the interface. Similarly, in the 17-4 PH/PLA/17-4 PH sandwich structure (Figure 8e,f), the best morphology was characterized by more compact contact and partial shear deformation, while in the low-performance case, interfacial smearing, poor fusion, and large void clusters showed a severe reduction in interlayer adhesion. It is known that in metal FFF “green part” layers, effective load carrying is limited due to the binder-dominant structure and natural porosity, and interfacial discontinuities act as sources of stress concentration, reducing mechanical strength. In general, layered architectures with homogeneous stacking and strong fusion are associated with high strength and more stable deformation, while debonding, buckling, and increased porosity have led to a significant decrease in mechanical performance.

### 3.3. RSM-Based Statistical Analysis and Optimization

The effects of manufacturing parameters on tensile strength were analyzed using a second-order RSM based on a general linear model (GLM) that allows for the simultaneous evaluation of continuous (infill ratio) and categorical (fill pattern and layer arrangement) factors [44]. The developed model showed high explanatory power, with R^2^ = 98.23%, Adj-R^2^ = 95.94, and Rpred^2^ = 86.46% indicating a strong fit with experimental data. ANOVA results (Table 3) show that the most dominant parameter on the UTS is the layer arrangement (Material configuration). This factor explained 85.82% of the total variance and was found to be statistically highly significant (*p* < 0.05). In contrast, the linear and quadratic terms of occupancy rate were not significant on their own (*p* > 0.05). However, the significant interaction between occupancy rate and layer arrangement (Infill × Material, *p* = 0.017) indicates that the occupancy effect varies depending on the hybrid structure type. The contribution of interactions related to occupancy rate and design remained limited and was found to be statistically insignificant. This finding is consistent with studies highlighting that in multi-material/layered FFF structures, the mechanical response is often controlled by load-bearing continuity paths and interface behavior. In particular, in multi-material FFF, interface compatibility and adhesion at the interface are key parameters determining ultimate strength and failure location [39]. The fact that infill percentage alone is not significant, while the Infill × Material interaction is significant, indicates that the effect of infill is a configuration-dependent effect. The literature reports that in single-material PLA samples, infill and/or infill geometry can significantly affect UTS, and this occurs via filament alignment and continuity load paths [45]. In this context, the shift in the “carrier” role from a purely “carrier” role to a more structured layer arrangement in hybrid layer architecture may have statistically weakened the occupancy effect while strengthening the interaction term, which makes the occupancy effect visible in specific arrangements.

The quadratic regression equations obtained for each material-pattern combination are presented in Table 4. These equations allow for the prediction of mechanical performance depending on the infill ratio and the numerical comparison of different production scenarios. Thus, the experimental design is supported by a mathematical prediction model.

**Figure 9:**
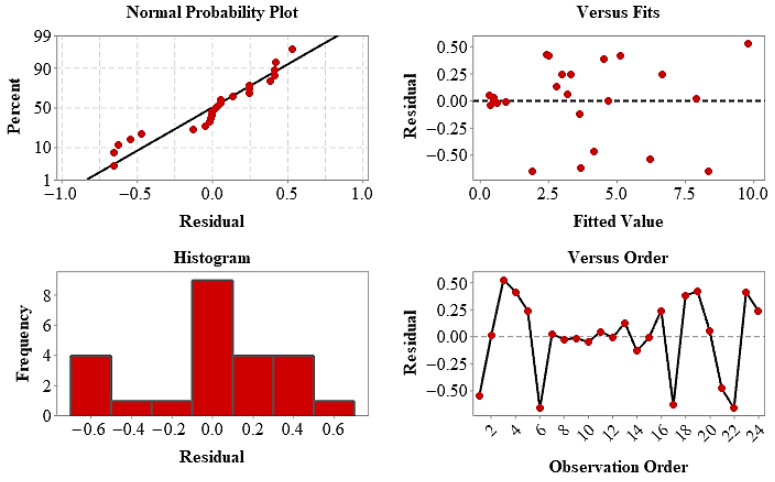
Residual diagnostic plots for UTS, including normal probability plot, residuals versus fits, histogram of residuals, and residuals versus observation order.

The statistical validity of the model has been confirmed by residual analyses. The normal distribution of residuals and their random scattering against the predicted values demonstrate that the model assumptions are met. The response surface and contour plots (Figure 9) visually confirm that the UTS is primarily controlled by the layer architecture, with infill ratio being a secondary optimization parameter. Finally, parameter combinations providing maximum tensile strength were determined using the Response Optimizer, and optimum production conditions were statistically predicted [46]. These findings demonstrate that layer arrangement is a key determinant of mechanical performance in hybrid composites, and that the RSM approach offers an effective and reliable tool for parameter optimization.

The model established to examine the effects of production parameters on yield strength showed good fit with experimental data, and the values R^2^ = 95.28%, Adj-R^2^ = 89.15%, and Rpred^2^ = 67.37% indicate that the model has sufficient explanatory and predictive capacity. ANOVA results (Table 5) show that the most effective parameter on yield strength is the layer arrangement (Material configuration). This factor explained 82.54% of the total variance and was found to be statistically significant (*p* = 0.009). In contrast, the linear and quadratic terms of infill ratio, the infill pattern, and the interactions between factors were found to be statistically insignificant (*p* > 0.05). This indicates that yield strength is primarily controlled by the hybrid layer architecture, while infill ratio and infill pattern have a secondary or limited effect. This situation can be explained by the fact that in hybrid/multi-material structures, yield initiation is often controlled by interface/layer transition regions and layer sequencing, which act as weak links. The finding that interface properties determine strength and failure initiation in multi-material FFF supports this mechanism [39]. The lack of a statistically significant difference between infill density and pattern on the yield strength indicates that in this hybrid architecture, the yield strength is shaped by the “layer configuration” rather than the “infill pattern”. This result is consistent with findings reporting that in multi-material/multi-layer panels, the mechanical response is predominantly controlled by the material sequence [47].

The quadratic regression equations obtained for each material-pattern combination are presented in Table 6. These equations allow for the estimation of yield strength depending on the infill ratio and the comparative evaluation of different production parameters.

**Figure 10:**
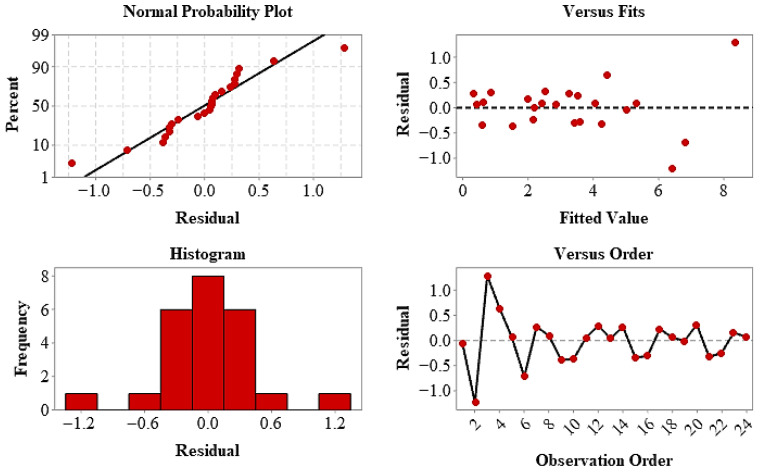
Residual diagnostic plots for YS, including normal probability plot, residuals versus fits, histogram of residuals, and residuals versus observation order.

The statistical fit of the model has been confirmed by residual analyses. The linear distribution of residuals in the normal probability plot and their random scattering against the predicted values indicate that the model assumptions are met and that there is no systematic deviation. The response surface and contour plots (Figure 10) visually support the fact that the yield strength (YS) varies largely depending on the layer arrangement and that the infill ratio offers a limited optimization effect. In conclusion, the layer configuration is the primary determinant of performance in terms of yield strength, and the RSM approach has been validated as an effective tool in modeling the mechanical behavior of hybrid structures and determining appropriate production parameters.

The model developed to examine the effects of manufacturing parameters on elongation to fracture showed a very high fit, with R^2^ = 98.64%, Adj-R^2^ = 96.87%, and Rpred^2^ = 92.01%, indicating that the experimental data were largely explained by the model. ANOVA results (Table 7) show that elongation to fracture is significantly affected by both layer arrangement and infill pattern. Layer arrangement was identified as the most dominant factor, explaining 71.90% of the total variance (*p* < 0.001), while infill pattern was found to be the second most influential factor with a contribution of 11.28% (*p* = 0.001). Furthermore, the linear term of the infill percentage is also statistically significant (*p* = 0.020), indicating that ductility is sensitive to the infill percentage [48,49]. In addition, the significant result of the Material × Pattern interaction (*p* = 0.001) reveals that elongation behavior is shaped not only by individual parameters but also by the combined effect of layer architecture and infill geometry. In contrast, the contribution of the quadratic term (Infill^2^) and other interactions remained limited. This result is expected from a mechanical perspective: tensile strength is often limited by “maximum load bearing” and interface damage, while elongation to fracture is more related to deformation paths, local buckling/tensile bonds, load transfer routes, and the “strain capacity” of the cellular architecture. It has been reported that infill geometry/mesh path can alter stress transfer and fracture mechanisms in tensile behavior, thus highlighting the pattern effect in ductility-sensitive metrics such as elongation [45,50]. In addition, it has been shown that in “sandwich/layered” structures, core geometry and core-crust bonding significantly influence deformation and energy absorption behavior [51,52]. In this context, the fact that the pattern for Elongation appears meaningful in your results supports the idea that shape change in the hybrid configuration can be controlled more effectively via the “inner padding architecture” and that this effect varies depending on the configuration (Material × Pattern).

The quadratic regression equations obtained for each material-pattern combination are presented in Table 8. These equations allow for the prediction of elongation behavior depending on the infill ratio and the comparison of different production configurations in terms of mechanical ductility.

**Figure 11:**
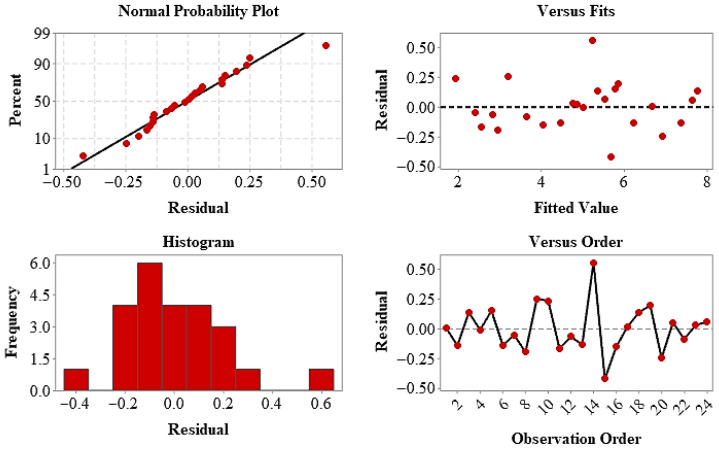
Residual diagnostic plots for EL, including normal probability plot, residuals versus fits, histogram of residuals, and residuals versus observation order.

The residuals now confirm that the model assumptions are met. The linear distribution of the residuals in the normal probability plot and their random scattering against the predicted values demonstrate that the normality and constant variance conditions are satisfied. The response surface and contour plots (Figure 11) visually support the finding that the elongation values vary significantly depending on the layer arrangement and infill geometry, and that ductility exhibits a multifactorial behavior. In conclusion, it has been determined that performance in terms of fracture elongation is controlled jointly by both structural architecture and infill geometry, and that the RSM approach is an effective method for quantitatively modeling these complex interactions and determining optimum parameters.

Overall, layer arrangement was determined to be the most dominant parameter for all three responses, while infill ratio and infill pattern showed secondary effects depending on the response type. The RSM approach has provided a reliable and effective tool for modeling the mechanical behavior of hybrid composites and determining optimum production conditions.

## 4. Conclusions

In this study, the mechanical performance of single and multi-material PLA/17-4 PH hybrid structures produced using an FDM-based additive manufacturing method was systematically investigated, considering the combined effects of layer arrangement, infill ratio, and infill pattern. Experimental results were quantitatively modeled using RSM. The findings clearly demonstrate that the mechanical behavior in multi-material FDM structures is controlled not only by volumetric infill parameters but primarily by layer architecture and interfacial load transfer mechanisms. Experimental results showed that the highest tensile and yield strengths were obtained in pure PLA samples with 100% infill ratio and linear fill geometry. This was attributed to the effectiveness of the linear raster structure, which provides increased material continuity, reduced porosity, and uninterrupted stress transfer in the load direction. In contrast, 17-4 PH “green part” samples without sintering exhibited the lowest mechanical performance due to the binder-dominant structure and limited interlayer adhesion. This result confirms the critical importance of sintering and densification steps for achieving the final metallic properties in metal FFF processes. Accordingly, this study specifically focused on the mechanical behavior of hybrid structures in the green state to isolate the effects of layer architecture and infill parameters on load transfer and structural response. Post-sintering properties, which involve additional phenomena such as densification, shrinkage, and microstructural evolution, were beyond the scope of the present work and are considered a direction for future research.

Multi-material hybrid configurations (PLA/17-4 PH/PLA and 17-4 PH/PLA/17-4 PH) showed intermediate performance in terms of mechanical properties. However, the layer order was found to be a determining factor in load-carrying capacity. The PLA–17-4 PH–PLA arrangement with PLA on the outer surfaces produced higher strength values due to more homogeneous stress distribution and more effective interlayer bonding. In the reverse configuration, stiffness mismatch and interlayer stress concentrations increased the tendency for delamination, leading to early failure. This finding demonstrates that mechanical performance optimization in hybrid structures can be achieved not only through material selection but also through functional layer arrangement. Fracture surface and cross-sectional morphology analyses supported the mechanical results from a microstructural perspective. High-performance samples showed strong filament fusion, low porosity, and a continuous layer architecture; while in low-performance structures, interlayer separation, void clustering, and weak interlayer bonding were identified as the dominant failure mechanisms. These observations clearly demonstrate that the primary determinant of strength in FDM structures is the quality of interlayer adhesion. Although the hybrid structures did not surpass the tensile strength of pure PLA in the green state, they provide a controllable architectural platform for integrating metal-rich regions within polymer-dominant structures, offering potential advantages in localized reinforcement and design-driven performance optimization. In particular, such hybrid architectures may be relevant for (i) green-state multi-material preforms intended for subsequent debinding and sintering processes, where controlled metal distribution is required, (ii) functionally graded structures demanding region-specific stiffness variation, and (iii) lightweight components incorporating metal-rich zones in selected load-bearing or wear-prone regions.

Statistical modeling results (R^2^ > 0.95) showed that the most dominant parameter affecting tensile and yield strength was the layer arrangement; a large portion (70–85%) of the mechanical variance was explained by this factor. The effects of infill ratio and infill pattern were secondary, but interaction terms were found to be significant in certain hybrid configurations. This indicates that the effect of infill in multi-material FDM systems is configuration-dependent and that design optimization should be performed considering holistic parameter interactions.

Overall, this study has experimentally and statistically demonstrated that:

- Layer arrangement is the primary design parameter in hybrid FDM structures;
- Infill density offers a secondary optimization tool by increasing mechanical continuity;
- Infill geometry plays a complementary role through the load path architecture;
- Interface adhesion and porosity control directly determine the final performance.

In conclusion, the proposed experimental–statistical approach provides a reliable design and optimization framework for multi-material FDM structures; it offers guiding information for the development of lightweight, functional, and customizable hybrid components. Future studies will further enhance the industrial applicability of hybrid FDM technology by investigating the effect of post-sintering density increase on the mechanical behavior of metal filaments, interfacial improvement strategies (chemical/thermal activation, surface roughening, etc.), and fatigue and environmental resistance performance under real service conditions.

## Figures and Tables

**Table 1:** Mechanical properties of PLA and 17-4 PH [21].

Properties	PLA	17-4 PH
Filament diameter (mm)	1.75	1.75
Specific gravity (g/cm^3^)	1.2	7.6
Tensile strength (MPa)	72	990
Elongation to fracture (%)	11.8	4
Print temperature (°C)	210–260	230–250
Bed Temperature (°C)	45–60	90–100
Print speed (mm/s)	40–100	15–50

**Table 2:** Process variables and their levels.

Parameters	Value
Nozzle size	0.4 mm
17-4 PH	270 °C
PLA	250 °C
Platform temperature (17-4 PH)	120 °C
Platform temperature (PLA)	60 °C
Platform temperature (17-4 PH/PLA/17-4 PH)	120 °C
Platform temperature (PLA/17-4 PH/PLA)	60 °C PLA, 90 °C 17-4 and 60 °C PLA
Layer height	0.15 mm
First layer height	0.25 mm
Base print speed	65 mm/s
Fill density	20/60/100%
Fill pattern	Line/Hexagon
Enable raft	No
Enable pre-extrusion	No
Enable wall	No
Back fan status	Always Off
Composite layer levels (Initial)	1.3 mm
Composite layer levels (Middle)	1.35 mm
Composite layer levels (Top)	1.35 mm

**Table 3:** Analysis of variance for UTS.

Source	DF	Seq SS	Contribution	Adj SS	Adj MS	F-Value	*p*-Value
Infill	1	12.516	7.44%	0.1948	0.19481	0.66	0.437
Infill^2^	1	0.054	0.03%	0.0535	0.05353	0.18	0.680
Material	3	144.337	85.82%	14.4292	4.80973	16.20	0.000
Pattern	1	1.875	1.12%	0.1317	0.13171	0.44	0.520
Infill × Material	3	4.953	2.94%	4.9527	1.65091	5.56	0.017
Infill × Pattern	1	0.110	0.07%	0.1104	0.11039	0.37	0.556
Material × Pattern	3	1.370	0.81%	1.3697	0.45657	1.54	0.265
Error	10	2.970	1.77%	2.9695	0.29695		
Total	23	168.184	100.00%				
Model Summary							
S	R-sq	R-sq(adj)	Press	R-sq(pred)	AICc	BIC	
0.544934	98.23%	95.94%	22.7687	86.46%	107.96	65.63	

**Table 4:** Regression equations for UTS.

Material	Pattern	Equation		
17-4 PH	Hexagonal	UTS	=	0.428 − 0.0058 × Infill + 0.000063 × Infill^2^
17-4 PH	Linear		=	0.461 − 0.0016 × Infill + 0.000063 × Infill^2^
17-4 PH/PLA17-4 PH	Hexagonal		=	1.645 + 0.0103 × Infill + 0.000063 × Infill^2^
17-4 PH/PLA17-4 PH	Linear		=	2.084 + 0.0145 × Infill + 0.000063 × Infill^2^
PLA	Hexagonal		=	4.418 + 0.0334 × Infill + 0.000063 × Infill^2^
PLA	Linear		=	5.439 + 0.0375 × Infill + 0.000063 × Infill^2^
PLA/17-4 PH/PLA	Hexagonal		=	2.693 + 0.0121 × Infill + 0.000063 × Infill^2^
PLA/17-4 PH/PLA	Linear		=	2.442 + 0.0163 × Infill + 0.000063 × Infill^2^

**Table 5:** Analysis of variance for YS.

Source	DF	Seq SS	Contribution	Adj SS	Adj MS	F-Value	*p*-Value
Infill	1	7.100	6.45%	0.0258	0.02580	0.05	0.828
Infill^2^	1	0.457	0.42%	0.4575	0.45747	0.88	0.370
Material	3	90.914	82.54%	10.6361	3.54536	6.82	0.009
Pattern	1	0.893	0.81%	0.1522	0.15219	0.29	0.600
Infill × Material	3	3.200	2.90%	3.1996	1.06653	2.05	0.171
Infill × Pattern	1	0.919	0.83%	0.9187	0.91872	1.77	0.213
Material × Pattern	3	1.462	1.33%	1.4622	0.48739	0.94	0.458
Error	10	5.198	4.72%	5.1978	0.51978		
Total	23	110.142	100.00%				
Model Summary							
S	R-sq	R-sq(adj)	Press	R-sq(pred)	AICc	BIC	
0.720959	95.28%	89.15%	35.9424	67.37%	121.39	79.06	

**Table 6:** Regression equations for YS.

Material	Pattern	Equation		
17-4 PH	Hexagonal	YS	=	0.867 − 0.0185 × Infill + 0.000183 × Infill^2^
17-4 PH	Linear		=	0.347 − 0.0065 × Infill + 0.000183 × Infill^2^
17-4 PH/PLA17-4 PH	Hexagonal		=	2.435 − 0.0184 × Infill + 0.000183 × Infill^2^
17-4 PH/PLA17-4 PH	Linear		=	2.235 − 0.0064 × Infill + 0.000183 × Infill^2^
PLA	Hexagonal		=	4.191+ 0.0080 × Infill + 0.000183 × Infill^2^
PLA	Linear		=	4.563+ 0.0200 × Infill + 0.000183 × Infill^2^
PLA/17-4 PH/PLA	Hexagonal		=	3.850 − 0.0164 × Infill + 0.000183 × Infill^2^
PLA/17-4 PH/PLA	Linear		=	2.865 − 0.0044 × Infill + 0.000183 × Infill^2^

**Table 7:** Analysis of variance for EL.

Source	DF	Seq SS	Contribution	Adj SS	Adj MS	F-Value	*p*-Value
Infill	1	6.5165	9.38%	0.72145	0.72145	7.64	0.020
Infill^2^	1	0.1396	0.20%	0.13964	0.13964	1.48	0.252
Material	3	49.9439	71.90%	9.24842	3.08281	32.66	0.000
Pattern	1	7.8364	11.28%	2.07111	2.07111	21.94	0.001
Infill × Material	3	0.5130	0.74%	0.51298	0.17099	1.81	0.209
Infill × Pattern	1	0.0132	0.02%	0.01317	0.01317	0.14	0.717
Material × Pattern	3	3.5533	5.12%	3.55335	1.18445	12.55	0.001
Error	10	0.9438	1.36%	0.94380	0.09438		
Model Summary							
S	R-sq	R-sq(adj)	Press	R-sq(pred)	AICc	BIC	
0.307214	98.64%	96.87%	5.54812	92.01%	80.45	38.12	

**Table 8:** Regression equations for EL.

Material	Pattern	Equations		
17-4 PH	Hexagonal	EL	=	1.491 + 0.0234 × Infill − 0.000101 × Infill^2^
17-4 PH	Linear		=	1.993 + 0.0220 × Infill − 0.000101 × Infill^2^
17-4 PH/PLA17-4 PH	Hexagonal		=	2.971 + 0.0358 × Infill − 0.000101 × Infill^2^
17-4 PH/PLA17-4 PH	Linear		=	5.224 + 0.0344 × Infill − 0.000101 × Infill^2^
PLA	Hexagonal		=	4.513 + 0.0273 × Infill − 0.000101 × Infill^2^
PLA	Linear		=	6.205 + 0.0258 × Infill − 0.000101 × Infill^2^
PLA/17-4 PH/PLA	Hexagonal		=	3.498 + 0.0287 × Infill − 0.000101 × Infill^2^
PLA/17-4 PH/PLA	Linear		=	3.967 + 0.0273 × Infill − 0.000101 × Infill^2^

## Data Availability

The original contributions presented in the study are included in the article. Further inquiries can be directed to the corresponding author.
